# Ferroptosis: a new strategy for cardiovascular disease

**DOI:** 10.3389/fcvm.2023.1241282

**Published:** 2023-09-04

**Authors:** Yuyuan Wang, Junduo Wu

**Affiliations:** Department of Cardiology, Second Hospital of Jilin University, Changchun, China

**Keywords:** ferroptosis, coronary artery disease, lipid peroxidation, regulatory cell death, ncRNAs

## Abstract

Cardiovascular disease (CVD) is currently one of the prevalent causes of human death. Iron is one of the essential trace elements in the human body and a vital component of living tissues. All organ systems require iron for various metabolic processes, including myocardial and skeletal muscle metabolism, erythropoiesis, mitochondrial function, and oxygen transport. Its deficiency or excess in the human body remains one of the nutritional problems worldwide. The total amount of iron in a normal human body is about 3–5 g. Iron deficiency may cause symptoms such as general fatigue, pica, and nerve deafness, while excessive iron plays a crucial role in the pathophysiological processes of the heart through ferroptosis triggered by the Fenton reaction. It differs from other cell death modes based on its dependence on the accumulation of lipid peroxides and REDOX imbalance, opening a new pathway underlying the pathogenesis and mechanism of CVDs. In this review, we describe the latest research progress on the mechanism of ferroptosis and report its crucial role and association with miRNA in various CVDs. Finally, we summarise the potential therapeutic value of ferroptosis-related drugs or ferroptosis inhibitors in CVDs.

## 1. Introduction

Cardiovascular diseases (CVDs) are a group of diseases involving the circulatory system. Statistically, they are responsible for about one-third of all fatalities globally. Examples of CVDs include ischemic heart disease, cardiomyopathy, valve disease, tachycardia, and heart failure, among others (1, 2). Cardiomyocyte mortality includes regulation cell death (RCD) and unintentional cell death (ACD) (3). While several RCD types, including apoptosis, autophagy, and pyroptosis, are associated with CVD pathogenesis, ferroptosis is a novel RCD modality. The ferroptosis inhibitor ferrostatin-1 has been shown to reduce doxorubicin (DOX)-induced cardiotoxicity and mortality in mice, whereas apoptosis, autophagy, and necrosis inhibitors had no effect (4). These findings demonstrate that ferroptosis, a distinct type of RCD, may contribute to the aetiology of CVD.

In 2002, Stockwell et al. demonstrated that the substance erastin results in cell mortality (5). Subsequently, in 2012, they defined ferroptosis as an erastin-induced cell death mechanism (6). They discovered that it lacked the hallmarks of apoptosis (chromatin marginalisation and condensation), autophagy (such as the creation of double-membrane closed vesicles), and necrosis (such as the swelling of organelles and the rupture of plasma membrane). Ferroptosis is characterised by mitochondrial shrinkage, increased membrane density, and the loss or absence of crests, with no discernible morphological alterations in the nucleus. In cellular components, ferroptosis is mainly characterised by an increase in reactive oxygen species (ROS) and lipid peroxidation (6). It has recently been linked to the pathogenesis of numerous illnesses, including cancer, nervous system diseases, and CVD (7, 8). Different cardiovascular conditions are associated with a rise in ROS, a hallmark of ferroptosis. Therefore, assessing the role of ferroptosis in different CVDs is crucial to improve their diagnosis and treatment. In this review, we discuss the function and potential regulatory mechanisms of ferroptosis in various CVDs.

## 2. Mechanisms underlying ferroptosis

**Figure 1:**
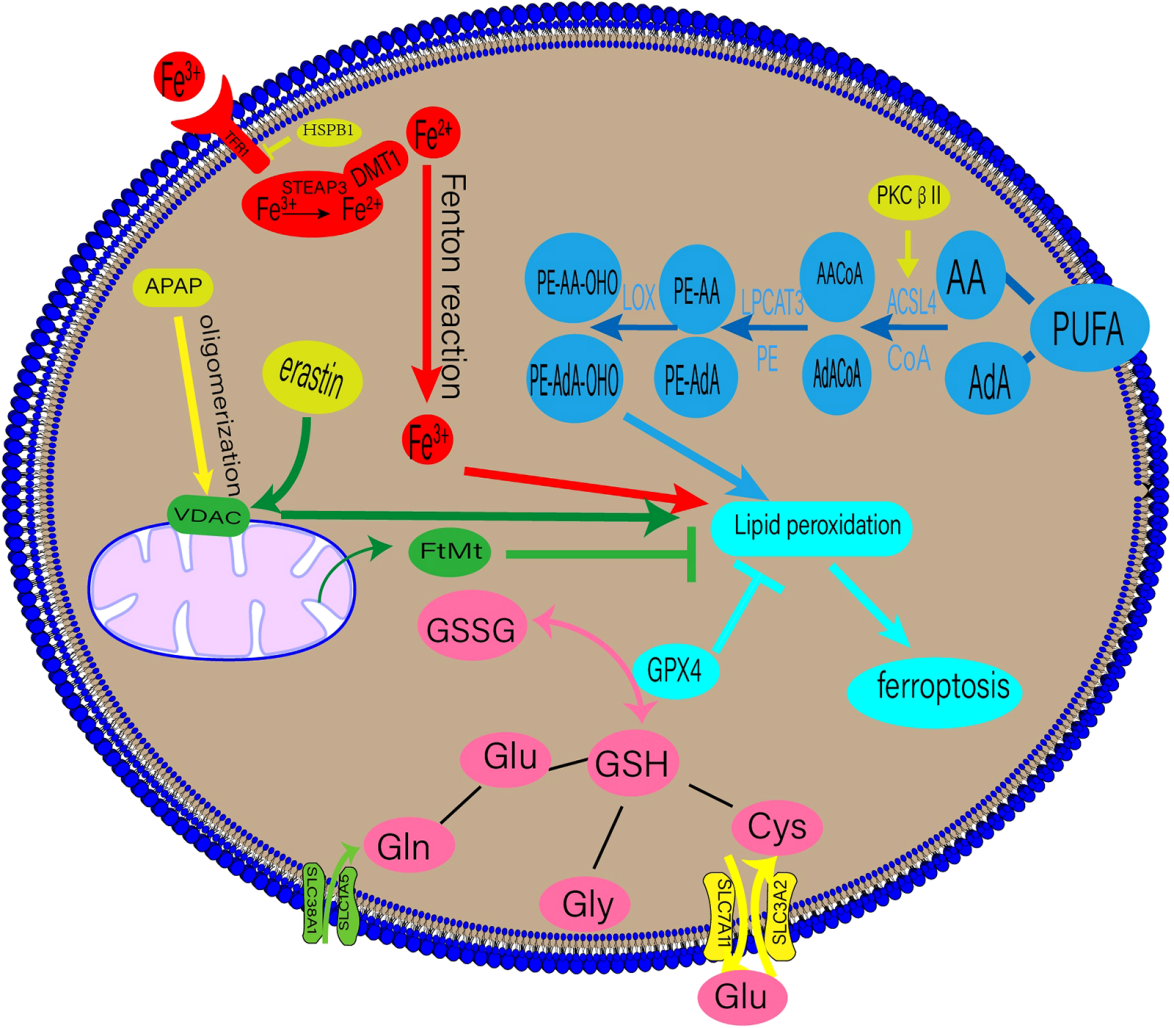
Regulatory mechanisms of ferroptosis. DMT1, divalent metal transporter 1; HSPβ1, Heat shock protein β1; STEAP3, six-transmembrane epithelial antigen of prostate 3; PUFAs, polyunsaturated fatty acids; AA, arachidonic acid; AdA, adrenal acid; ACSL4, long-chain fatty acyl-CoA synthase 4; LPCAT3, lysolecithin acyltransferase 3; LOXs, lipoxygenases; GPX4, glutathione peroxidase 4; GSSG, oxidized glutathione; GSH, glutathione; TF, transferrin; TFR1, transferrin receptor 1; APAP, acetaminophen; FtMt, mitochondrial ferritin.

Ferroptosis is accompanied by an imbalance in the production and removal of free radicals, which may be related to aberrant lipid, amino acid, iron, or mitochondrial metabolism. The underlying mechanisms of ferroptosis are shown in Figure 1.

### 2.1. Lipid metabolism and ferroptosis

Lipid peroxidation is a crucial stage of ferroptosis. Any lipid peroxidation-related substance can control ferroptosis (6). The main building blocks of cell membranes are polyunsaturated fatty acids (PUFAs), which are readily peroxidised during ferroptosis. Lipid peroxidation can be classified into enzymatic and non-enzymatic. When iron enters the cell via transferrin (TF), it undergoes the Fenton reaction with H2O2 to produce an excess of oxygen-free radicals, which mainly powers the non-enzymatic process. Lipid degradation and ferroptosis can be caused by free radicals and PUFA interaction (9).

An enzymatic response involves the induction of ferroptosis and ROS production using numerous enzymes. Crucial enzymes in this process include lysolecithin acyltransferase 3 (LPCAT3) and long-chain fatty acyl-CoA synthetase 4 (ACSL4) (9). Arachidonic acid (AA) and adrenal acid (AdA) can be converted into the fatty acyl coenzymes AA-CoA and AdA-CoA, respectively, by the enzyme ACSL4 (10). LPCAT3 then esterifies AA-CoA and AdA-CoA to create phosphatidylethanolamine (PE)-AA and PE-AdA, which is then oxidised by lipoxygenase (LOX) to produce the hazardous lipid hydroperoxides (LOOH) products. The enhanced expression of PE-AA-OHO and PE-AdA-OHO is associated with ferroptosis (11). Recent research by Zhang et al. suggests that phosphorylating ACSL4 may accelerate the accumulation of lipid peroxidation products, intensifying ferroptosis (12).

### 2.2. Iron metabolism and ferroptosis

Iron, a necessary human body component, is primarily used to produce haemoglobin and myoglobin. It is involved in numerous physiological processes, such as oxygen transfer and storage, mitochondrial respiration, and intracellular enzymatic reactions (13). These processes depend on balanced iron metabolism, with iron overload and iron deficiency leading to pathological illnesses. Iron-dependant lipid peroxidation is closely associated with ferroptosis (9). The bulk of the body's iron requirement is met by the external diet or the physiological breakdown of ageing red blood cells. TF on the cell membrane binds extracellular Fe3+, producing TF-Fe3+, which is then absorbed by the cell through transferrin receptor 1 (TFR1). Intracellular Fe3+ is converted to Fe2+ in the endosome of the six-transmembrane epithelial antigen of prostate 3 (STEAP3) and released into the cytosol by the divalent metal transporter 1(DMT1) (14, 15). Fe2+ is stored in the ferritin molecules and released by ferroportin 1(FPN1). When the dynamic balance between iron intake, use, and recycling is disrupted, free Fe2+ combines with H2O2 to form a lipid peroxide with a high oxidative capacity, resulting in ferroptosis (14).

Several iron metabolism-related proteins and regulating elements can influence ferroptosis. TFR1 is an iron transporter involved in iron metabolism. Iron regulatory protein (IRP) and hypoxia-inducible factor-1 (HIF-1) increase the expression of TFR1, raising cellular iron absorption and susceptibility to ferroptosis. Notably, reduced iron consumption prevents ferroptosis (16). Additionally, without TFR1, the metal cation transporter ZIP14, or SLC39A14, can function as an iron transporter, bringing iron into the cell and causing ferroptosis (17).

Furthermore, a significant relationship exists between ferroptosis and autophagy. Nuclear receptor coactivator 4 (NCOA4) is a ferritin breakdown receptor. Controlling ferritin phagocytosis can help maintain the intracellular iron balance (18). NCOA4-dependent ferritin autophagy increases the cell's vulnerability to ferroptosis (19). CISD1 and CISD2 are crucial for mitochondrial iron transfer, and CISD1 suppression increases iron-mediated mitochondrial lipid peroxidation and erastin-dependent ferroptosis (20). Contrarily, CISD2 inhibition increases the levels of ferrous iron and lipid ROS in the mitochondria, accelerating sulfasalazine-mediated ferroptosis (21). These results suggest that iron metabolism plays a significant role in ferroptosis.

### 2.3. Amino acid metabolism and ferroptosis

A contributing factor to ferroptosis is glutathione (GSH), which comprises glutamate, cysteine, and glycine via peptide bonds in response to catalytic enzymes. It is present in almost all human tissue cells (22). Glutaminase 2 (GLS2) primarily converts glutamine (Gln) into glutamate, and Gln enters cells through a receptor comprising SLC38A1 and SLC1A5. Exogenous cysteine entering the cell via the glutamate cystine antiporter (XC-), consisting of SLC3A2 and SLC7A11, is converted primarily into cysteine. GSH is present in reduced (GSH) and oxidised disulfide (S-S-S) forms, both of which have strong antioxidant properties. Oxidised glutathione (GSSG) has antioxidant and antiatherogenic effects on lipoproteins and macrophages when enclosed in liposomes (23). Glutathione peroxidase 4 (GPX4) converts GSH into GSSG in the presence of radicals, and toxic lipid hydrogen peroxide generated by the cells into nontoxic lipid alcohols (24). A drop in GSH levels leads to ferroptosis from an accumulation of ROS and lipid peroxides.

### 2.4. Mitochondria and ferroptosis

Mitochondria are the chief energy source for many metabolic processes, such as the tricarboxylic acid cycle and oxidative phosphorylation. ROS are primarily produced by mitochondrial oxidative phosphorylation. During ferroptosis, mitochondrial changes, such as a decrease in its volume and an increase in its membrane density, are seen. The relationship between mitochondria and ferroptosis has received much attention recently (25). Iron regulation depends on mitochondrial ferritin (FtMt). Mice lacking FtMt exhibit severe neurological impairments, reversed by FtMt overexpression (26). A mitochondria-targeting antioxidant called MitoTEMPO was found to protect mice with adriamycin-induced cardiomyopathy from cardiac damage caused by ferroptosis (4). Atorvastatin induces a dose-dependent decrease in the viability of human cardiomyocytes and mouse skeletal muscle cells, increasing intracellular ROS levels and lipid peroxidation. These changes mainly affect the mitochondria, resulting in their malfunction (27).

Furthermore, the voltage-dependent anion channel (VDAC) on the outer mitochondrial membrane has been shown to mediate ferroptosis (28). VDAC regulates mitochondrial metabolism by increasing ion and molecule exchange between the cytoplasm and mitochondria (28). As early as 2007, VDAC was recognised as a potential erastin target (29). Acetaminophen (APAP) overdoses can lead to ferroptosis in hepatocytes. Hepatocytes exposed to APAP showed VDAC1 oligomerisation, and VBIT-12, an inhibitor of the oligomerisation, reduced ferroptosis, treating mitochondrial malfunction (30).

## 3. Ferroptosis and CVD

Intravenous iron supplementation has been shown to improve the apnoea-hypopnoea index (AHI) in patients with anaemia, iron deficiency, heart failure and sleep-disordered breathing, improving their quality of life (31). However, ferroptosis caused by excess iron is closely related to various CVDs. Non-coding RNAs (ncRNAs) play a crucial role in CVDs and are useful noninvasive diagnostic markers (32). Here, we present a general review of the recent studies that suggest the role of ncRNAs in CVD-related ferroptosis (Table 1).

**Table 1:** Ferroptosis-related ncRNAs in cardiovascular disease.

NcRNA	Target	Effect	Disease	References
miR-23a-3p	SLC7A11	Promote ferroptosis	Atrial fibrillation	(33)
miR-190a-5p	GLS2	Inhibit ferroptosis	Myocardial infarction	(34)
miR-15a-5p	GPX4	Promote ferroptosis	Myocardial infarction	(35)
miR-23a-3p	DMT1	Inhibit ferroptosis	Myocardial infarction	(36)
lncRNA-Gm47283/miR-706	Ptgs2	Promote ferroptosis	Myocardial infarction	(37)
circRNA1615/miR-152-3p	LRP6	Inhibit ferroptosis	Myocardial infarction	(38)
miR-149	HMGB1	Inhibit ferroptosis	Septic cardiomyopathy	(39)
miR-140-5p	SLC7A11	Promote ferroptosis	Obesity-induced cardiac injury	(40)
lncRNA KCNQ1OT1/miR-7-5p	–	Promote ferroptosis	DOX-induced cardiac injury	(41)
lncRNA-ZFAS1 /miR-150-5p	CCND2	Promote ferroptosis	Diabetic cardiomyopathy	(42)
miR-375-3p	GPX4	Promote ferroptosis	Heart failure	(43)
circSnx12/miR-224-5p	–	–	Heart failure	(44)

### 3.1. Ferroptosis and oxidative stress

Oxidative stress is the result of an imbalance between oxidation and antioxidation processes. The ROS levels in the human body vary in a manageable way under metabolic circumstances. However, the body's ability to balance oxidation and antioxidation is disrupted in response to harmful stimuli, leading to ROS accumulation and cell damage or death (45). Oxidative stress is closely associated with inflammation. It can be exacerbated by inflammation, triggering transcription factors, such as Nrf2, NF-B1, and pro-inflammatory cytokines like TNF, altering the production of inflammatory cytokines and anti-inflammatory molecules (46). Recent research has revealed a new inflammatory program of cell death, called PANoptosis, was discovered, which has key features of pyroptosis, apoptosis, and necroptosis, but cannot be characterized by any of the three alone. According to Tong et al., mice with metabolic dysfunction-associated fatty liver disease (MAFLD), which may be associated with PANoptosis regulation, can be treated by LPT1, a ferroptosis inhibitor, suggesting a relation between ferroptosis and PANoptosis (47).

Ferroptosis is primarily characterised by an increase in lipid peroxides and ROS, disrupting the cellular oxidation-reduction processes, resulting in cell death. GSH and GPX4, major regulators of ferroptosis, are also implicated in controlling the inflammatory response. GSH functions as an antioxidant, preventing cell damage by buffering unusually high ROS levels (48). GPX4 reduces inflammation by eliminating oxidative byproducts of AA catabolism (49). Another key modulator of antioxidants is the nuclear factor erythroid 2-related factor 2 (Nrf2). Under oxidative stress, Nrf2 binds to antioxidant response elements in the nucleus, initiating the transcription of antioxidant genes and defending the cells from cytotoxic and oxidative harm (50). In addition, Nrf2 plays a role in ferroptosis by controlling several signalling pathways. Several lipid peroxide-inhibiting proteins and enzymes, including GPX4, SLC40A1, and SLC48A1, are encoded by Nrf2 target genes and mitigate ferroptosis (51).

### 3.2. Ferroptosis and immune modulation

The occurrence of cardiovascular disease is closely related to changes in the body's immune function. Coronary atherosclerosis is essentially an inflammatory response process, and efferocytosis is the process by which macrophages remove diseased or dead cells from blood vessels, which is important for maintaining homeostasis in the environment within tissues. When the inflammatory response is disrupted, the decomposition effect is inhibited, and these lesions or dead cells accumulate in the blood vessels to form plaques, eventually causing coronary atherosclerosis, myocardial infarction, and stroke, among others (52). CD47-SIRP*α* plays an important role in regulating the immune system, and CD47 can bind to the SIRP*α* protein on the surface of immune cells to inhibit immune cells from phagocytosing cancer cells. Notably, inhibiting CD47 expression can block this process, promote the cleanup of dead cells by macrophages, and reduce plaque accumulation in blood vessels (53). Recently, Zhai et al. invented a DOX delivery nanoplatform called p-LDM, which reduces DOX toxicity and increases its efficacy. This platform promotes ferroptosis, leading to better inhibition of tumour cell proliferation. However, marked enhancement of ferroptosis was observed after a combination of p-LDM and CD47-SIRPα blockade, which may be related to the up-regulation of interferon-γ (IFN-γ) level and the inhibition of cystine/glutamate antitransporter (system Xc-) mediated by CD47-SIRPα blockade(54, 55).

Additionally, CD8+ T cells can reduce the expression level of system Xc- subunits SLC3A2 and SLC7A11 by releasing IFN-γ, which promotes the ferroptosis of tumour cells (56). In atherosclerosis, CD8+ T cells can also play both atheroprotective and pro-atherogenic roles. CD8+ T cells can be recruited to the vessel wall and play a role in the inflammatory response (57). However, how CD8+ T cells regulate ferroptosis and contribute to atherosclerosis remains poorly understood.

### 3.3. Ferroptosis and dietary iron overload

Haemoglobin and the biological operation of humans both depend on iron, a vital element. However, in rodent models, excess iron intake has been shown to worsen liver damage by raising serum triglyceride and cholesterol levels (58). Excess iron can also exacerbate atherosclerosis (AS) by accumulating in the arterial wall. It worsens the atherosclerotic process by altering vascular permeability and lipid profile and elevating pro-atherosclerotic inflammatory mediators (59). A meta-analysis revealed a significant relationship between high dietary haem iron intake and an increased risk of cardiovascular death (60). Thus, lowering the consumption of iron-containing meals may help lower the risk of developing CVD.

### 3.4. Ferroptosis and coronary heart disease

AS is primarily a fatty metabolic disorder, and ferroptosis possibly contributes to its development and progression. Guo et al. have reported that the overexpression of GPX4, a ferroptosis regulator, reduces vascular cell sensitivity to oxidised lipids delaying the progression of AS (61). Coronary AS is the most prevalent form of AS. Based on biological information analysis, Wu et al. have identified seven ferroptosis-related genes, including CA9, CBS, CEBPG, HSPB1, SLC1A4, STMN1, and TRIB3, that are associated with ischemic heart disease (62). Modulating immune responses, amino acid metabolism, and numerous pathways implicated in the pathogenesis of Coronary heart disease (CHD) may also play a part in its regulation (62).

The first event in the pathogenesis of AS is endothelial cell damage caused by lipid peroxidation-induced ferroptosis (63). Previous research has demonstrated that tanshinone IIA (TSA) inhibits AS by significantly decreasing ROS and lipid ROS levels produced in response to ferroptosis inducers in human cardiac endothelial cells while restoring GSH function. TSA inhibits ferroptosis by elevating Nrf2 mRNA (63). Nrf2 has also been related to ferroptosis in human coronary endothelial cells. Moreover, the isoprene diphosphate synthase subunit (PDSS2) is responsible for the formation of AS (64). Yang et al. have shown that AS patients have lower serum levels of PDSS2 and Nrf2, and overexpression of PDSS2 reduces iron concentration and ferroptosis in human coronary endothelial cells (HCAECs) while increasing HCAECs proliferation (64). However, the curative effects of PDSS are blunted because Nrf2 mRNA is downregulated in HCAECs. Overexpression of PDSS2 suppresses ferroptosis in HCAECs by activating the Nrf2 pathway (64).

The mitochondrial transporter GLS2 controls the conversion of Gln to glutaminase. Zhou et al. have shown that GLS2 overexpression in mice with myocardial infarction (MI) increases cardiomyocyte ferroptosis. GLS2 is blocked by miR-190a-5p preventing ferroptosis (34). Additionally, miR-15a-5p is thought to be involved in the ferroptosis of cardiomyocytes. GPX4 is a direct target of miR-15a-5p, and in a mouse model of MI, miR-15a-5p overexpression enhanced ferroptosis. The transcription factor early growth response 1 (Egr-1) increases GPX4 synthesis while decreasing miR-15a-5p levels, preventing cardiomyocyte ferroptosis (35).

Exosomes made from human umbilical cord blood mesenchymal stem cells (HUCB-MSC) were found to have cardioprotective effects in a rat model of acute MI (AMI). By transporting Fe2+, the divalent metal transporter 1 (DMT1) improves iron metabolism, and DMT1 overexpression results in cardiomyocyte ferroptosis. HUCB-MSC carries miR-23a-3p and prevents the synthesis of DMT1 and ferroptosis (36).

Additionally, downstream miRNA can be regulated by lncRNA and circRNA to aid in the process of ferroptosis in MI (37, 38). LncRNA Gm47283 specifically targets miR-706 and PTGS2, known ferroptosis regulators. In a rat model of MI, overexpression of the lncRNA Gm47283 suppressed miR-706 expression to increase PTGS2 level and ferroptosis damage, a process reversed by siRNA within the stem cell membrane (37).

### 3.5. Cardiomyopathy

Cardiomyopathy is an organic heart disorder with multiple potential causes. Nonetheless, the genetic cause is substantially the most prevalent. Mechanical or electrical heart dysfunction caused by cardiomyopathy can lead to heart failure and, eventually, cardiac death (65). Despite the different causes, all forms of cardiomyopathy are associated with elevated levels of ROS and lipid peroxidation (66), suggesting a robust relationship between cardiomyopathy and ferroptosis.

#### 3.5.1. DOX-induced cardiomyopathy

The anticancer drug adriamycin is associated with dose-dependent cardiotoxicity. DOX-induced cardiomyopathy includes cardiomyocyte structural abnormalities in response to adriamycin (67). In 2014, Ichikawa found a significant increase in the mitochondrial iron levels in the myocardial tissue of patients with DOX-induced cardiomyopathy compared to those with normal cardiac function or other types of cardiomyopathy, which was reversed by dexrazoxane (68). According to Tadokoro et al. ferroptosis is the primary cause of cell death in DOX-induced cardiomyopathy. Mitochondria-dependent ferroptosis is caused by excessive lipid peroxidation via the DOX-Fe2+ complex, with downregulation of GPX4 expression (69). In addition, MITOL/MARCH5 controls the number of mitochondria and their function. Kitakata et al. have shown that the knockdown of MITOL leads to reduced GPX4 mRNA in the mitochondria and ferroptosis in rat cardiomyocytes. Lipid peroxidation and ferroptosis are significantly reduced in MITOL mutant cells upon GPX4 activation (70). However, PRMT4 binds with Nrf2 to increase its enzymatic methylation, thereby limiting Nrf2 nuclear translocation, decreasing GPX4 transcription, and enhancing DOX-induced cardiac ferroptosis (67).

Methyltransferase-like 14 (METTL14), an m6A writing protein, is widely involved in the progression of CVD (71). By facilitating the m6A modification of lncRNA KCNQ1OT1, METTL14 reduces the activity of miR-7-5p. Reduced miR-7-5p expression leads to higher transferrin receptor levels, increased iron uptake, and higher lipid ROS production. Consequently, selective inhibition of the METTL14/KCNQ1OT1/miR-7-5p axis provides a unique method for preventing DOX-induced cardiac damage by negatively mediating cardiomyocyte ferroptosis (41).

#### 3.5.2. Septic cardiomyopathy

Extreme cases of septic cardiomyopathy (SCM), a cardiac dysfunction caused by septic shock, can result in heart failure and mortality. Studies have indicated that ferroptosis may contribute to cardiac damage in patients with sepsis (72). Gong et al. have used bioinformatics to pinpoint the roles of Cdkn1a, Ptgs2, Nfe2l2, Rela, and Vim5 in modulating ferroptosis in SCM (72). The inflammatory reaction is primarily regulated by islet cell autoantigen 69 (ICA69). In addition to ICA69, inflammatory cytokines, ROS, and ferroptosis markers are upregulated in the myocardium of LPS-treated mice. ICA69 knockdown significantly reduced the expression of ferroptosis markers, suggesting the potential of targeting ICA69 as a therapeutic strategy for septic cardiomyopathy (73).

#### 3.5.3. Diabetic cardiomyopathy

Diabetic cardiomyopathy is diagnosed in individuals with diabetes when coronary AS and hypertension do not account for abnormalities in the myocardial structure or function (74). Diabetic cardiomyopathy may be regulated in part by Nrf2 (75). Long-term diabetes inhibits autophagy, reducing Nrf2-mediated defence and activating Nrf2-regulated pathogenic processes, resulting in increased lipid peroxidation, cardiomyocyte ferroptosis, and heart muscle deterioration, accelerating the development of diabetic cardiomyopathy (75). Nrf2 inhibition reverses the pathological changes, apoptosis, and oxidative stress in the hearts of patients with type I diabetes. It also reduces the development and severity of diabetic cardiomyopathy.

Palmitic acid (PA)-induced cardiac damage is one of the major contributors to type 2 diabetes mellitus (T2DM)-related cardiomyopathy (76). Wang et al. have shown that PA triggers cell death in H9C2 cardiomyocytes by decreasing the protein levels of heat shock factor 1 (HSF1) and GPX4 (76), which were restored by ferroptosis inhibitors. Furthermore, HSF1 overexpression restored PA-suppressed GPX4 expression, inhibiting PA-induced cardiomyocyte ferroptosis (76).

In a mouse model of diabetic cardiomyopathy, lncRNA-ZFAS1 increased cardiomyocyte death and ferroptosis. It inhibited miR-150-5p expression and cyclin D2 (CCND2) production in diabetic cardiomyopathy, enhancing cardiomyocyte ferroptosis. Therefore, inhibiting lncRNA-ZFAS1 may be a strategy for managing and avoiding diabetic cardiomyopathy (42).

#### 3.5.4. Hypertensive cardiomyopathy

A leading cause of heart failure is hypertensive heart disease, which results from long-term untreated hypertension and causes abnormalities in the structure and function of the heart. Microvascular endothelial cells (CMVECs) in the heart are rich in the apelin receptor, which binds to the new endogenous ligand elabela. In rodents, angiotensin II (Ang II) can significantly reduce elabela levels. In mice with Ang II-induced hypertension, administration of elabela or ferrostatin-1 significantly attenuated the myocardial remodelling and ultrastructural damage. Elabela decreases Ang II-induced iron content and lipid peroxidation by increasing cardiac xCT/GPX4 signalling and inhibiting IL-6/STAT3 signalling. Hence, elabela can potentially delay the progression of hypertensive heart disease by inhibiting Ang II-mediated CMVEC ferroptosis and ventricular restructuring (77).

#### 3.5.5. Hypertrophic cardiomyopathy

Fang et al. have shown that the cardiomyocytes of ferritin H-deficient animals have lower iron concentrations and higher oxidative stress (78). However, rodents with larger hearts maintained an iron-rich diet. In addition, ferroptosis worsens due to decreased GSH levels in cardiomyocytes and elevated lipid peroxidation. SLC7A11 overexpression was found to increase GSH levels and decrease cardiac ferroptosis in subsequent studies (78). Ptgs2, malondialdehyde, and ROS levels substantially increase Ang II-induced cardiac hypertrophy; however, ferroptosis blocker xCT attenuates these changes, inhibiting cardiac hypertrophy (79).

#### 3.5.6. Sickle cell disease-induced cardiomyopathy

Sickle cell disease (SCD), characterised by massive haemolysis, results in excess haem in the plasma leading to CVD (80). Ferroptosis is a vital mechanism underlying SCD cardiomyopathy. The process of haem breakdown can produce free iron by haem oxygenase 1 (Hmox1). In the SCD mouse model, cardiomyopathy worsened as the heme levels increased, simultaneously elevating ferroptosis marker levels. Hence, inhibiting or activating Hmox1 could reduce or increase myocardial ferroptosis, whereas a ferroptosis inhibitor could improve cardiomyopathy in mice (80).

### 3.6. Atrial fibrillation

The mechanism underlying iron-induced arrhythmia remains unknown. Previous studies have shown that rats with chronic iron overload develop heart block, longer PR intervals, and atrial fibrillation (AF) (81). Increased ROS production caused by iron overload leads to mitochondrial malfunction and changes in membrane potential, which can be one of the primary reasons for developing arrhythmia (82). Researchers have shown that excessive alcohol consumption can activate ferroptosis and increase the likelihood of AF. However, iron overload, ROS production, and AF susceptibility can all be reduced by inhibiting ferroptosis (83). Sepsis is a risk factor for the onset of AF, and sepsis-related myocardial injury has been associated with ferroptosis (84). Fang et al. have shown that in the atria of septic rats, the expression of transferrin FPN, the only identified mammalian non-haem iron export protein, is significantly down-regulated. FPN knockdown significantly increased the intracellular iron concentration, increasing susceptibility to AF, which was reversed by suppressing ferroptosis. This finding demonstrates that in patients with recently diagnosed AF, LPS-induced endotoxemia may be influenced by ferroptosis (85).

AF is mainly caused by myocardial fibrosis. Liu et al. have shown that ferroptosis-related proteins in H9C2 cells undergo a series of sequential changes, including a gradual decrease in the expression of SLC7A11 and GPX4 in a canine model of fast atrial pacing. Exosomes from cardiac fibroblasts also make H9C2 cells more ferroptotic. Moreover, Liu et al. later discovered that in H9C2 cells, rapid pacing significantly increased the expression of miR-23a-3p, and SLC7A11 was a target gene. The damage to H9C2 cells was lessened following therapy with a miR-23a-3p inhibitor(33). Therefore, miRNA intervention is a potential strategy to prevent AF recurrence of AF by reducing ferroptosis and oxidative damage (33).

### 3.7. Pulmonary arterial hypertension

Studies have shown that iron deficiency is present in 43%–63% of people with pulmonary arterial hypertension and is associated with decreased exercise tolerance and higher mortality (86). Pulmonary vascular remodelling is the main pathogenic factor in pulmonary hypertension. In mice with chronic iron deficiency, pulmonary vascular cells switched to aerobic glycolysis, leading to pulmonary vascular remodelling through inflammatory cell infiltration and mitochondrial malfunction (87). Zou et al. identified seven genes, including BCL2, GCLM, MSMO1, SLC7A11, SRXN1, TSPA5, and TXNRD1, through biological data analysis that may be involved in regulating iron metabolism (86). Among the most crucial elements in pulmonary vascular remodelling are the suppression of cell death and abnormal proliferation of pulmonary artery smooth muscle cells (PASMCs) (88). Overexpression of SLC7A11 in PASMCs prevents ferroptosis, promotes proliferation, and ultimately accelerates pulmonary vascular remodelling. Erastin can induce ferroptosis by reducing the expression of GPX4 and SLC7A11, inhibiting the proliferation of hypoxic PASMCs both *in vitro* and *in vivo* (88).

### 3.8. Viral myocarditis

The most common enterovirus and the main culprit behind viral myocarditis is coxsackievirus. Kung et al. have shown that acyl-CoA synthetase long-chain family member 4 (ACSL4), the primary cause of ferroptosis, is involved in forming viral replication organelles. Enteroviruses cause ferroptosis through ACSL4, suggesting its potential as a therapeutic target for viral myocarditis (89).

### 3.9. Heart failure

The final stage of many cardiovascular disorders is heart failure, involving myocardial fibrosis and cardiac remodelling. Iron anomalies (both deficiency and overload) in cardiomyocytes are closely associated with heart failure. Moreover, ferritin H is essential for maintaining iron homeostasis in the heart. Ferritin H-deficient mice showed reduced Slc7a11 expression in cardiac cells, while selective overexpression of Slc7a11 increased GSH levels and prevented cardiac ferroptosis (78, 90). These findings suggest that ferritin H is essential for avoiding heart failure. In addition, the adipose tissue macrophages (ATMs) express high levels of miR-140-5p in their exosomes, which may contribute to obesity caused by a long-term high-fat diet. miR-140-5p can inhibit the synthesis of GSH and encourage ferroptosis in cardiomyocytes by targeting SLC7A11 (40).

Through bioinformatics analysis, Chen et al. have shown that ferroptosis and autophagy are related to TLR4 and NADPH oxidase 4 (NOX4). The generation of superoxide anion, hydrogen peroxide, and cardiomyocyte ferroptosis are stimulated when TLR4 binds to NOX4. Knockdown of TLR4 and NOX4 in the heart by siRNA lentivirus in HF rats reduced cardiomyocyte death and enhanced ventricular remodelling. Their loss also delayed the activation of autophagy and ferroptosis in HF rats. Therefore, TLR4-NOX4 may be a potential therapeutic target for heart failure (91).

The principal causes of heart failure following an ischemia-reperfusion (I/R) insult are ventricular remodelling and eventual cardiac fibrosis. In rat cardiomyocytes, miR-375-3p increased myocardial fibrosis and accelerated the progression of heart failure by enhancing cardiomyocyte ferroptosis through GPX4 control (43). In the myocardium of HF mice, circSnx12 acted as an endogenous sponge for miR-224-5p. Notably, miR-224-5p also has a binding site in the 3'-UTR region of FTH1. Consequently, miR-224-5p may be a circSnx12 target in heart failure-related ferroptosis (44).

### 3.10. Heart transplantation

Following a heart transplant, I/R damage may cause aseptic inflammation, ultimately lowering the transplant's success rate. The majority of patients who undergo cardiac transplantation have advanced heart disease. Aseptic inflammation is mainly caused by neutrophil recruitment. Through the TLR4/Trif/type I IFN pathway, ferroptosis enhances the adhesion of coronary vascular endothelial cells to neutrophils. While ferrostatin-1(Fer-1), a ferroptosis inhibitor, has no impact on neutrophil recruitment to the inflammatory site, it can significantly prevent neutrophils and endothelial cells from adhering to the walls of the coronary arteries. Thus, inhibiting the ferroptosis process may improve the prognosis of patients with heart transplants (92).

### 3.11. Aortic aneurysm and dissection

An aortic aneurysm is a prevalent condition with a high fatality rate. It is characterised by smooth muscle cell (SMC) damage or loss, elastic fibre destruction, aortic wall weakness, dilatation, and dissection. Currently, no treatments can clinically halt the progression of aortic aneurysm and dissection. Hence, to effectively treat this condition, it is critical to explore its molecular mechanisms (93)Recently, Zheng et al. screened 40 differential genes from a ferroptosis-related genes (FRG) dataset in abdominal aortic aneurysms (AAAs) and normal samples. GPX4 was found to be a key gene involved in AAA-related ferroptosis. TNF, NOD-like signal pathways, and ferroptosis-related immune cell infiltration also play a key role in AAA (94). PCSK9 plays an essential role in lipid metabolism, and its inhibitors have been widely used clinically to reverse atherosclerotic plaques and reduce LDL cholesterol. PCSK9 is highly expressed in AAA tumour necks and regulates ferroptosis by inducing ROS production, mitochondrial dysfunction, and regulating lipid metabolism (95).

## 4. Ferroptosis and CVD treatment

**Figure 2:**
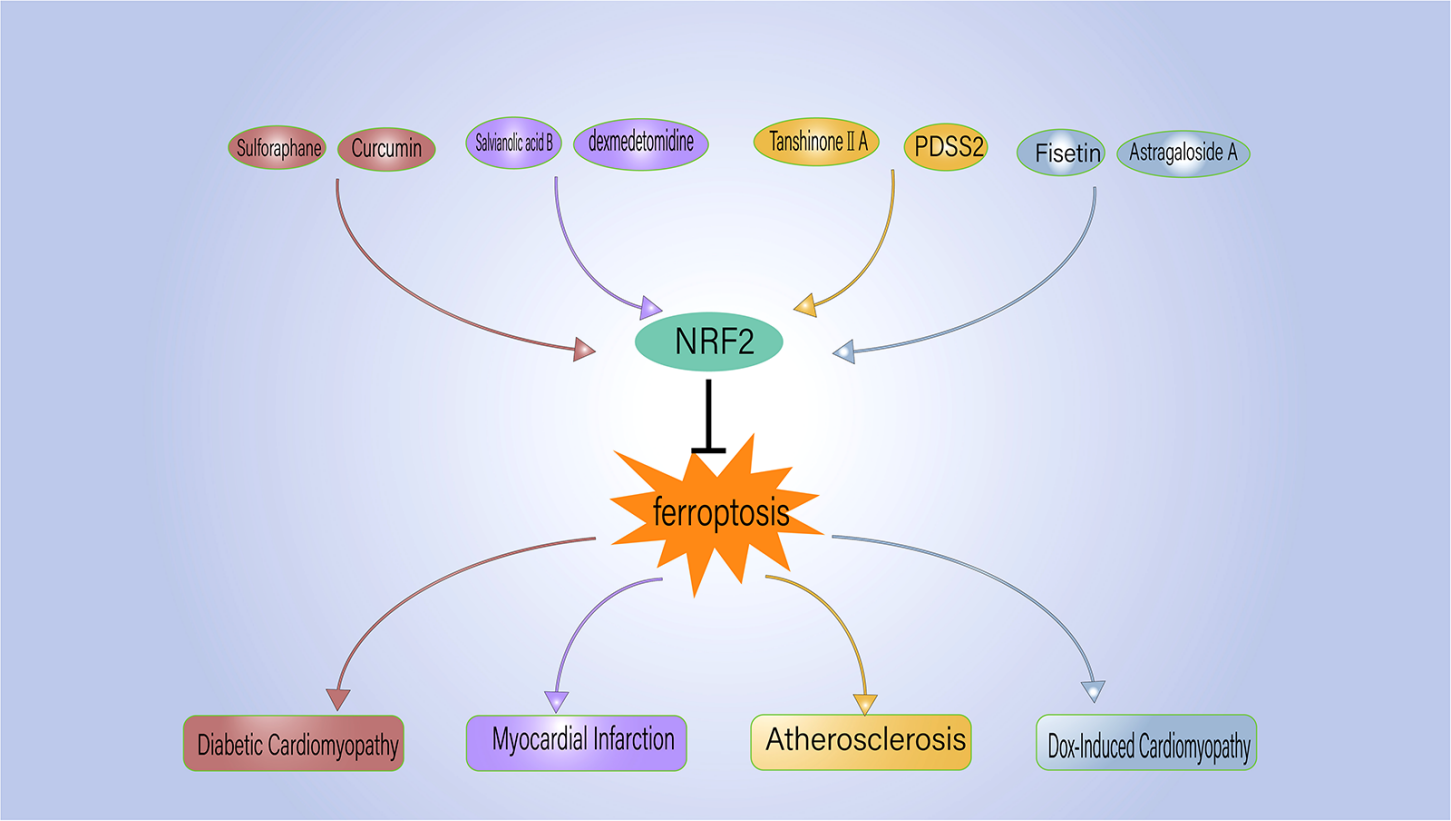
NRF2 regulates the ferroptosis process associated with cardiovascular disease. PDSS2, prenyldiphosphate synthase subunit 2.

Given the strong association between ferroptosis and several CVDs, active research has been ongoing to find ferroptosis inhibitors that can prevent and treat CVD (Table 2). Ferrostatin-1 and deferoxamine (DFO) are some ferroptosis inhibitors under evaluation. Nrf2, a regulator of ferroptosis and antioxidants, is also crucial to the development and progression of CVD. Hence, controlling Nrf2 levels is another therapeutic strategy to prevent/inhibit ferroptosis (Figure 2).

**Table 2:** Small- molecule modulators and clinical drugs to target ferroptosis in cardiovascular diseases.

Drug	Mechanism	Target	References
Fer-1	Reduces the production of ROS and Inhibits lipid peroxidation	Diabetic myocardial; ischemia/reperfusion; heart transplantation	(92, 96)
Atorvastatin	Decrease the mRNA levels of PTGS2, contents of malonaldehyde and protein levels of NOX4 and increase the contents of GSH	Heart failure	(97)
Canagliflozin	Regulated autophagy	Heart failure	(98)
Dexmedetomidine	Activation of Nrf2 through AMPK/GSK-3β signaling pathway	Myocardial ischemia/reperfusion injury	(99)
C3G	Downregulated TfR1 expression, and upregulated the expressions of FTH1 and GPX4	Myocardial ischemia/reperfusion injury	(100)
Melatonin	Regulates YAP	Dox-induced cardiotoxicity	(101)
Resveratrol	Regulation of ubiquity specific peptidase 19 (USP19)-Beclin1 autophagy	Myocardial ischemia/reperfusion injury	(102)
	Inhibits the production of HMGB1 by upregulating miR-149	Septic cardiomyopathy	(39)
DFO	–	Myocardial ischemia/reperfusion injury	(103)
Britanin	Upregulate GPX4	Myocardial ischemia/reperfusion injury	(104)
BRD4770	Inhibit the inflammatory response and lipid peroxidation	Aortic dissection	(105)
Salvianolic acid B	Activates NRF2	Myocardial infarction	(106)
MSC-EVs	Reduce the release of NETs by turning NETosis into apoptosis	Abdominal aortic aneurysm	(107)

### 4.1. Ischemia-reperfusion injury

Fer-1 is a first-generation ferroptosis inhibitor (6) that suppresses lipid ROS production and inhibits erastin or RSL3-induced ferroptosis. It improves cardiac function in animal models of diabetes and I/R damage (96). Additionally, Fer-1 increases the longevity of patients with heart transplants by preventing neutrophil recruitment after cardiac cell death (92). Cyanidin-3-glucoside (C3G), a member of the anthocyanin family of antioxidants, frequently found in red or purple fruits and vegetables, has anti-inflammatory, antioxidant, and heart-protective properties (108). Studies in a rat model have demonstrated that C3G protects against cardiac I/R injury. Rat H9C2 cells treated with C3G showed a drop in Fe2+ levels and TFR1 expression. It blocks the deubiquitylation of Beclin1 through K11, inhibiting ferroptosis (100). Therefore, C3G might be useful for preventing I/R myocardial injury.

Inula sinensis yields britanin (Bri), a bioactive substance with potent antioxidant and anti-inflammatory properties (109). Lu et al. found that Bri suppresses apoptosis and ferroptosis and reduces the size of cardiac infarcts in mice following I/R (104). The Food and Drug Administration of the United States has approved the use of the iron chelator DFO to treat conditions caused by an excess of iron (110). DFO lowers the lipid peroxides in heart tissue averting myocardial damage in a rat model of I/R injury (103). Resveratrol is a naturally occurring bioactive polyphenol with anti-inflammatory, anticancer, and antioxidant properties. I/R injury causes ferroptosis in the cardiac cell model of H9C2 rats with I/R, resulting in higher levels of oxidative stress and Fe2+ than in normal cells. Resveratrol also inhibits autophagy, mediated by ubiquitin-specific peptidase 19 (USP19)-Beclin1, to minimise ferroptosis (102). Dexmedetomidine reduces iron accumulation and lipid peroxidation caused by hypoxia/reoxygenation in cardiomyocytes through Nrf2 activation via AMPK/GSK-3 signalling, inhibiting myocardial ferroptosis (99).

### 4.2. DOX-induced cardiomyopathy

DOX-induced cardiomyopathy is closely associated with the Nrf2 pathway, with several pathway members contributing to DOX-induced cardiac ferroptosis (67, 111, 112). Fisetin has been shown to significantly inhibit DOX-induced cardiotoxicity in rats by reversing the reduction in GPX4 levels and lowering myocardial fibrosis, cardiac hypertrophy, and cardiomyocyte ferroptosis. It also increases the expression of SIRT1, Nrf2 mRNA and protein levels and its nuclear translocation to safeguard the myocardium (112). Furthermore, intravenous astragaloside decreased DOX-induced cardiac ferroptosis by activating the Nrf2 signalling pathway and increasing GPX4 expression (111).

Ethoxyquinoline, a lipophilic antioxidant often used in food preservation, suppresses ferroptosis caused by GPX4 deficiency in DOX-induced cardiomyopathy by lowering DOX-induced increases in malondialdehyde and mitochondrial lipid peroxidases. Although its effect on apoptosis is not immediately obvious, ethoxyquinoline is a potential therapeutic that could prevent DOX-induced ferroptosis in cardiomyopathy (113).

Salidroside significantly reduces the fibrosis and heart malfunction caused by adriamycin *in vivo*. By limiting iron accumulation, restoring GPX4-dependent antioxidant activity, and preventing cellular or mitochondrial lipid peroxidation, it inhibits ferroptosis *in vivo* and *in vitro* (114). Melatonin is a mitochondrial antioxidant, mainly used to improve sleep quality. However, it also safeguards the heart. It has been shown to prevent DOX-induced ferroptosis by markedly upregulating ACSL4, a ferroptosis-related protein and downregulating GPX4. By increasing YAP protein levels, melatonin can decrease DOX-induced ferroptosis and cardiotoxicity (101).

### 4.3. Diabetic cardiomyopathy

Isoadiponectin (ISO) has been shown to increase microvessel density and perfusion, promote MFN2 overexpression in diabetic mice, and inhibit mitochondria-associated ferroptosis to protect heart microvessels in patients with diabetes (115).

Historically, sulforaphane and curcumin were thought to have anticancer and antioxidant properties (116). Ferroptosis in diabetic cardiomyopathy can be mitigated through Nrf2 modulation using sulforaphane and curcumin (117, 118). Sulforaphane inhibits ferroptosis in rodents with diabetic cardiomyopathy by increasing ferritin and SLC7A11 levels and stimulating Nrf2 through AMP-activated protein kinase (AMPK) (117). Curcumin attenuates glucose-induced ferroptosis in diabetic rabbit cardiomyocytes by increasing Nrf2 nuclear translocation, increasing the expression of oxidative scavenging factors like HO-1, and reducing the loss of GPX4 (118). These results suggest novel therapeutic alternatives for the management of diabetic cardiomyopathy.

### 4.4. Septic cardiomyopathy

LPS is a well-known contributor to heart dysfunction in septic cardiomyopathy. Catalase, an antioxidant enzyme, inhibits LPS-induced heart dysfunction by controlling multiple processes, including ferroptosis, oxidative stress, autophagy, and apoptosis (119). Resveratrol, an anti-inflammatory and antioxidant, has been shown to play a role in cardiomyocyte ferroptosis. In a mouse model of endotoxemia, resveratrol therapy improves GSH levels and decreases lipid ROS, lipid peroxidation, and iron accumulation in cardiomyocytes. It inhibits the production of HMGB1 by upregulating miR-149, which reduces the ferroptosis of endotoxemia cardiomyocytes (39).

### 4.5. Myocardial infarction

Salvia miltiorrhiza contains salvianolic acid B, a strong antioxidant. Salvinolic acid B is found in rats with myocardial infarction, similar to Fer-1. It reverses ferroptosis, including lipid peroxide accumulation, mitochondrial damage, and expression of ferroptosis-related proteins, activating the Nrf2 signalling pathway and preventing myocardial infarction (106).

Geniposide (GEN), the main active ingredient of Gardeniae fructus, has natural antioxidant qualities and protects the heart. In rats with myocardial infarction, it reduced lipid peroxidation and iron overload, preventing ischemic heart damage (120).

### 4.6. Aortic aneurysm and dissection

Aortic aneurysm and dissection are detected based on the loss of smooth muscle cells. Ferroptosis-related markers, such as TFR, HOMX1, and ferritin, are noticeably higher in individuals with aortic dissection. BRD4770 functions as a histone methyltransferase inhibitor, preventing the mono-, dio-, and trimethylation of histone H3 lysine 9 (H3K9me1/2/3). System Xc-GPX4, FSP1-CoQ10, and GCH1-BH4 reduced morbidity and mortality in a mouse model of aortic dissection by reactivating the canonical ferroptosis pathways inhibited by ferroptosis inducers (105).

The formation of neutrophil extracellular traps (NETs), composed of depolymerised chromatin and intracellular granulin in the centrioles, can trap and kill pathogens by neutrophil death, or NETosis.NETosis has been shown to cause ferroptosis in smooth muscle cells (SMCs) by blocking the PI3K/AKT pathway and inducing the formation of AAA. Extracellular vesicles made from mesenchymal stem cells (MSC-EVs) can delay the formation of AAA by converting NETosis into apoptosis and reducing the release of NETs (107).

### 4.7. Heart failure

Atorvastatin has long been used as a treatment for coronary heart disease. It inhibits the activation of ferroptosis-related signals in a mouse model of isoproterenol (ISO)-induced heart failure by decreasing the levels of PTGS2 (a ferroptosis marker) mRNA, malondialdehyde, and NOX4 protein and increasing the GSH levels. Additionally, it defends the myocardium (97).

Sodium-glucose cotransporter 2 inhibitors (SGLT2i) have recently been found to provide significant cardiovascular benefits in patients with heart failure with preserved ejection fraction (HFpEF). Ma et al. have shown that ferroptosis plays a vital role in the HFpEF rat model induced by a high-salt diet, and canagliflozin protects against heart failure by reducing ferroptosis (98). SGLT2i decreases ferritin and hepcidin while increasing the transferrin receptors, thereby lowering the iron levels that help treat chronic heart failure. The mechanisms underlying this effect are based on the “cytoplasmic iron consumption” and “cytoplasmic iron supplementation” hypotheses. The former postulates that SGLT2i increases erythropoietin-related iron utilisation, leading to cytoplasmic Fe2+ reduction, while the latter contends that functional iron insufficiency is promoted by the inflammatory response in some patients with mild to moderate heart failure. SGLT2i inhibits hepcidin and ferritin by directly activating the sirtuin-1 signalling pathway, eliminating the need for intravenous iron therapy. It improves aberrant iron utilisation and treats functional iron deficiency (121). Therefore, clinical trials are required to confirm if iron supplementation therapy is necessary for treating heart failure with SGLT2i.

## 5. Conclusions and perspectives

Ferroptosis, characterised by lipid peroxidation, has been associated with numerous CVDs and promoted as a potential directions of treatment. It differs from other cell death processes because of the accumulation of intracellular iron, lipid peroxides, and mitochondrial alterations. Its role in the onset and progression of several diseases has been well studied.

Drugs inhibiting ferroptosis by reducing lipid peroxidation and iron accumulation can affect the development and progression of CVDs. Additionally, ncRNAs have been shown to play a crucial role in CVDs and are useful noninvasive biomarkers. Ferroptosis can be effectively controlled by reducing the expression of related ncRNAs, offering a unique therapeutic strategy for treating CVDs.

However, there are several challenges to treating CVD and ferroptosis together. First, research is warranted on the specific processes and pathways relating ferroptosis to CVDs to find new treatments. Second, there is little clinical research focused on human subjects since most current studies on ferroptosis and CVDs are in cells or animals. More clinical trials must be performed to support the use of different ferroptosis inhibitors for CVDs. The ferroptosis inhibitors currently in clinical practice are likely to have unidentified adverse effects on other organs, including the liver and kidney, which need to be explored.

## References

[1] Vergallo R Crea F. Atherosclerotic plaque healing. N Engl J Med. (2020) 383(9):846–57. 10.1056/NEJMra2000317 32846063

[2] Arnett DK Blumenthal RS Albert MA Buroker AB Goldberger ZD Hahn EJ 2019 Acc/Aha guideline on the primary prevention of cardiovascular disease: executive summary: a report of the American college of cardiology/American heart association task force on clinical practice guidelines. Circulation. (2019) 140(11):e563–95. 10.1161/cir.0000000000000677 30879339 PMC8351755

[3] Tang D Kang R Berghe TV Vandenabeele P Kroemer G. The molecular machinery of regulated cell death. Cell Res. (2019) 29(5):347–64. 10.1038/s41422-019-0164-5 30948788 PMC6796845

[4] Fang X Wang H Han D Xie E Yang X Wei J Ferroptosis as a target for protection against cardiomyopathy. Proc Natl Acad Sci U S A. (2019) 116(7):2672–80. 10.1073/pnas.1821022116 30692261 PMC6377499

[5] Dolma S Lessnick SL Hahn WC Stockwell BR. Identification of genotype-selective antitumor agents using synthetic lethal chemical screening in engineered human tumor cells. Cancer Cell. (2003) 3(3):285–96. 10.1016/s1535-6108(03)00050-3 12676586

[6] Dixon SJ Lemberg KM Lamprecht MR Skouta R Zaitsev EM Gleason CE Ferroptosis: an iron-dependent form of nonapoptotic cell death. Cell. (2012) 149(5):1060–72. 10.1016/j.cell.2012.03.042 22632970 PMC3367386

[7] Yan HF Zou T Tuo QZ Xu S Li H Belaidi AA Ferroptosis: mechanisms and links with diseases. Signal Transduct Target Ther. (2021) 6(1):49. 10.1038/s41392-020-00428-9 33536413 PMC7858612

[8] Del Re DP Amgalan D Linkermann A Liu Q Kitsis RN. Fundamental mechanisms of regulated cell death and implications for heart disease. Physiol Rev. (2019) 99(4):1765–817. 10.1152/physrev.00022.2018 31364924 PMC6890986

[9] Sun Y Xue Z Huang T Che X Wu G. Lipid metabolism in ferroptosis and ferroptosis-based cancer therapy. Front Oncol. (2022) 12:941618. 10.3389/fonc.2022.941618 35978815 PMC9376317

[10] Cheng J Fan YQ Liu BH Zhou H Wang JM Chen QX. Acsl4 suppresses glioma cells proliferation via activating ferroptosis. Oncol Rep. (2020) 43(1):147–58. 10.3892/or.2019.7419 31789401 PMC6912066

[11] Dixon SJ Winter GE Musavi LS Lee ED Snijder B Rebsamen M Human haploid cell genetics reveals roles for lipid metabolism genes in nonapoptotic cell death. ACS Chem Biol. (2015) 10(7):1604–9. 10.1021/acschembio.5b00245 25965523 PMC4509420

[12] Zhang HL Hu BX Li ZL Du T Shan JL Ye ZP Pkcβii phosphorylates Acsl4 to amplify lipid peroxidation to induce ferroptosis. Nat Cell Biol. (2022) 24(1):88–98. 10.1038/s41556-021-00818-3 35027735

[13] Fan X Li A Yan Z Geng X Lian L Lv H From iron metabolism to ferroptosis: pathologic changes in coronary heart disease. Oxid Med Cell Longev. (2022) 2022:6291889. 10.1155/2022/6291889 35993022 PMC9385341

[14] Hu H Chen Y Jing L Zhai C Shen L. The link between ferroptosis and cardiovascular diseases: a novel target for treatment. Front Cardiovasc Med. (2021) 8:710963. 10.3389/fcvm.2021.710963 34368260 PMC8341300

[15] Chen Z Yan Y Qi C Liu J Li L Wang J. The role of ferroptosis in cardiovascular disease and its therapeutic significance. Front Cardiovasc Med. (2021) 8:733229. 10.3389/fcvm.2021.733229 34765653 PMC8576275

[16] Zhang Y Xin L Xiang M Shang C Wang Y Wang Y The molecular mechanisms of ferroptosis and its role in cardiovascular disease. Biomed Pharmacother. (2022) 145:112423. 10.1016/j.biopha.2021.112423 34800783

[17] Yu Y Jiang L Wang H Shen Z Cheng Q Zhang P Hepatic transferrin plays a role in systemic iron homeostasis and liver ferroptosis. Blood. (2020) 136(6):726–39. 10.1182/blood.2019002907 32374849 PMC7414596

[18] Mancias JD Wang X Gygi SP Harper JW Kimmelman AC. Quantitative proteomics identifies Ncoa4 as the cargo receptor mediating ferritinophagy. Nature. (2014) 509(7498):105–9. 10.1038/nature13148 24695223 PMC4180099

[19] Fang X Ardehali H Min J Wang F. The molecular and metabolic landscape of iron and ferroptosis in cardiovascular disease. Nat Rev Cardiol. (2023) 20(1):7–23. 10.1038/s41569-022-00735-4 35788564 PMC9252571

[20] Yuan H Li X Zhang X Kang R Tang D. Cisd1 inhibits ferroptosis by protection against mitochondrial lipid peroxidation. Biochem Biophys Res Commun. (2016) 478(2):838–44. 10.1016/j.bbrc.2016.08.034 27510639

[21] Kim EH Shin D Lee J Jung AR Roh JL. Cisd2 inhibition overcomes resistance to sulfasalazine-induced ferroptotic cell death in head and neck cancer. Cancer Lett. (2018) 432:180–90. 10.1016/j.canlet.2018.06.018 29928961

[22] Forman HJ Zhang H Rinna A. Glutathione: overview of its protective roles, measurement, and biosynthesis. Mol Aspects Med. (2009) 30(1–2):1–12. 10.1016/j.mam.2008.08.006 18796312 PMC2696075

[23] Rosenblat M Volkova N Coleman R Aviram M. Anti-oxidant and anti-atherogenic properties of liposomal glutathione: studies in vitro, and in the atherosclerotic apolipoprotein E-deficient mice. Atherosclerosis. (2007) 195(2):e61–8. 10.1016/j.atherosclerosis.2007.05.012 17588583

[24] Bajic VP Van Neste C Obradovic M Zafirovic S Radak D Bajic VB Glutathione “redox homeostasis” and its relation to cardiovascular disease. Oxid Med Cell Longev. (2019) 2019:5028181. 10.1155/2019/5028181 31210841 PMC6532282

[25] Balaban RS Nemoto S Finkel T. Mitochondria, oxidants, and aging. Cell. (2005) 120(4):483–95. 10.1016/j.cell.2005.02.001 15734681

[26] Wang P Cui Y Ren Q Yan B Zhao Y Yu P Mitochondrial ferritin attenuates cerebral ischaemia/reperfusion injury by inhibiting ferroptosis. Cell Death Dis. (2021) 12(5):447. 10.1038/s41419-021-03725-5 33953171 PMC8099895

[27] Zhang Q Qu H Chen Y Luo X Chen C Xiao B Atorvastatin induces mitochondria-dependent ferroptosis via the modulation of Nrf2-Xct/Gpx4 axis. Front Cell Dev Biol. (2022) 10:806081. 10.3389/fcell.2022.806081 35309902 PMC8927716

[28] Lipper CH Stofleth JT Bai F Sohn YS Roy S Mittler R Redox-dependent gating of VDAC by mitoNEET. Proc Natl Acad Sci U S A. (2019) 116(40):19924–9. 10.1073/pnas.1908271116 31527235 PMC6778226

[29] Yagoda N von Rechenberg M Zaganjor E Bauer AJ Yang WS Fridman DJ RAS-RAF-MEK-dependent oxidative cell death involving voltage-dependent anion channels. Nature. (2007) 447(7146):864–8. 10.1038/nature05859 17568748 PMC3047570

[30] Niu B Lei X Xu Q Ju Y Xu D Mao L Protecting mitochondria via inhibiting VDAC1 oligomerization alleviates ferroptosis in Acetaminophen-induced acute liver injury. Cell Biol Toxicol. (2022) 38(3):505–30. 10.1007/s10565-021-09624-x 34401974

[31] Caravita S Faini A Vignati C Pelucchi S Salvioni E Cattadori G Intravenous iron therapy improves the hypercapnic ventilatory response and sleep disordered breathing in chronic heart failure. Eur J Heart Fail. (2022) 24(10):1940–9. 10.1002/ejhf.2628 35867685 PMC9804720

[32] Poller W Dimmeler S Heymans S Zeller T Haas J Karakas M Non-coding RNAs in cardiovascular diseases: diagnostic and therapeutic perspectives. Eur Heart J. (2018) 39(29):2704–16. 10.1093/eurheartj/ehx165 28430919 PMC6454570

[33] Liu D Yang M Yao Y He S Wang Y Cao Z Cardiac fibroblasts promote ferroptosis in atrial fibrillation by secreting exo-miR-23a-3p targeting SLC7A11. Oxid Med Cell Longevity. (2022) 2022:3961495. 10.1155/2022/3961495 PMC9168132 35677105

[34] Zhou X Zhuo M Zhang Y Shi E Ma X Li H. miR-190a-5p regulates cardiomyocytes response to ferroptosis via directly targeting GLS2. Biochem Biophys Res Commun. (2021) 566:9–15. 10.1016/j.bbrc.2021.05.100 34111670

[35] Fan K Huang W Qi H Song C He C Liu Y The Egr-1/miR-15a-5p/GPX4 axis regulates ferroptosis in acute myocardial infarction. Eur J Pharmacol. (2021) 909:174403. 10.1016/j.ejphar.2021.174403 34339707

[36] Song Y Wang B Zhu X Hu J Sun J Xuan J Human umbilical cord blood-derived mscs exosome attenuate myocardial injury by inhibiting ferroptosis in acute myocardial infarction mice. Cell Biol Toxicol. (2021) 37(1):51–64. 10.1007/s10565-020-09530-8 32535745

[37] Gao F Zhao Y Zhang B Xiao C Sun Z Gao Y Suppression of lncRNA Gm47283 attenuates myocardial infarction via miR-706/Ptgs2/ferroptosis axis. Bioengineered. (2022) 13(4):10786–802. 10.1080/21655979.2022.2065743 35485136 PMC9208485

[38] Li RL Fan CH Gong SY Kang S. Effect and mechanism of LRP6 on cardiac myocyte ferroptosis in myocardial infarction. Oxid Med Cell Longev. (2021) 2021:8963987. 10.1155/2021/8963987 34712388 PMC8548150

[39] Wang X Simayi A Fu J Zhao X Xu G. Resveratrol mediates the miR-149/HMGB1 axis and regulates the ferroptosis pathway to protect myocardium in endotoxemia mice. Am J Physiol Endocrinol Metab. (2022) 323(1):E21–e32. 10.1152/ajpendo.00227.2021 35532075

[40] Zhao X Si L Bian J Pan C Guo W Qin P Adipose tissue macrophage-derived exosomes induce ferroptosis via glutathione synthesis inhibition by targeting SLC7A11 in obesity-induced cardiac injury. Free Radic Biol Med. (2022) 182:232–45. 10.1016/j.freeradbiomed.2022.02.033 35271999

[41] Zhuang S Ma Y Zeng Y Lu C Yang F Jiang N METTL14 promotes doxorubicin-induced cardiomyocyte ferroptosis by regulating the KCNQ1OT1-miR-7-5p-TFRC axis. Cell Biol Toxicol. (2023) 39(3):1015–35. 10.1007/s10565-021-09660-7 34648132

[42] Ni T Huang X Pan S Lu Z. Inhibition of the long non-coding RNA ZFAS1 attenuates ferroptosis by sponging miR-150-5p and activates CCND2 against diabetic cardiomyopathy. J Cell Mol Med. (2021) 25(21):9995–10007. 10.1111/jcmm.16890 34609043 PMC8572773

[43] Zhuang Y Yang D Shi S Wang L Yu M Meng X MiR-375-3p promotes cardiac fibrosis by regulating the ferroptosis mediated by GPX4. Comput Intell Neurosci. (2022) 2022:9629158. 10.1155/2022/9629158 35498204 PMC9054412

[44] Zheng H Shi L Tong C Liu Y Hou M. circSnx12 is involved in ferroptosis during heart failure by targeting miR-224-5p. Front Cardiovasc Med. (2021) 8:656093. 10.3389/fcvm.2021.656093 33969020 PMC8097164

[45] Violi F Pignatelli P Basili S. Nutrition, supplements, and vitamins in platelet function and bleeding. Circulation. (2010) 121(8):1033–44. 10.1161/circulationaha.109.880211 20194876

[46] Yu Y Yan Y Niu F Wang Y Chen X Su G Ferroptosis: a cell death connecting oxidative stress, inflammation and cardiovascular diseases. Cell Death Discov. (2021) 7(1):193. 10.1038/s41420-021-00579-w 34312370 PMC8313570

[47] Tong J Lan XT Zhang Z Liu Y Sun DY Wang XJ Ferroptosis inhibitor liproxstatin-1 alleviates metabolic dysfunction-associated fatty liver disease in mice: potential involvement of panoptosis. Acta Pharmacol Sin. (2023) 44(5):1014–28. 10.1038/s41401-022-01010-5 36323829 PMC10104837

[48] Mak TW Grusdat M Duncan GS Dostert C Nonnenmacher Y Cox M Glutathione primes T cell metabolism for inflammation. Immunity. (2017) 46(4):675–89. 10.1016/j.immuni.2017.03.019 28423341

[49] Li C Deng X Xie X Liu Y Friedmann Angeli JP Lai L. Activation of glutathione peroxidase 4 as a novel anti-inflammatory strategy. Front Pharmacol. (2018) 9:1120. 10.3389/fphar.2018.01120 30337875 PMC6178849

[50] Wang YWJ Xiang Y Wang RQ Li XN Wang JX Yu SW Sulforaphane enhances Nrf2-mediated antioxidant responses of skeletal muscle induced by exhaustive exercise in HIIT mice. Food Sci Hum Wellness. (2022) 11(5):1355–61. 10.1016/j.fshw.2022.04.035

[51] Dodson M Castro-Portuguez R Zhang DD. Nrf2 plays a critical role in mitigating lipid peroxidation and ferroptosis. Redox Biol. (2019) 23:101107. 10.1016/j.redox.2019.101107 30692038 PMC6859567

[52] Doran AC Jr Yurdagul A Tabas I. Efferocytosis in health and disease. Nat Rev Immunol. (2020) 20(4):254–67. 10.1038/s41577-019-0240-6 31822793 PMC7667664

[53] Singla B Lin HP Ahn W Xu J Ma Q Sghayyer M Loss of myeloid cell-specific SIRPα, but not CD47, attenuates inflammation and suppresses atherosclerosis. Cardiovasc Res. (2022) 118(15):3097–111. 10.1093/cvr/cvab369 34940829 PMC9732525

[54] Kong X He Z Zhang Y Fang Y Liu D Wu H Intelligent self-amplifying ferroptosis-inducible nanoplatform for enhanced tumor microenvironment reconstruction and combination therapy. Chem Eng J. (2023) 468:143729. 10.1016/j.cej.2023.143729

[55] He Z Zhou H Zhang Y Du X Liu S Ji J Oxygen-boosted biomimetic nanoplatform for synergetic phototherapy/ferroptosis activation and reversal of immune-suppressed tumor microenvironment. Biomaterials. (2022) 290:121832. 10.1016/j.biomaterials.2022.121832 36228518

[56] Wang W Green M Choi JE Gijón M Kennedy PD Johnson JK CD8(+) T cells regulate tumour ferroptosis during cancer immunotherapy. Nature. (2019) 569(7755):270–4. 10.1038/s41586-019-1170-y 31043744 PMC6533917

[57] Schäfer S Zernecke A. CD8(+) T cells in atherosclerosis. Cells. (2020) 10(1):37. 10.3390/cells10010037 33383733 PMC7823404

[58] Fujiwara S Izawa T Mori M Atarashi M Yamate J Kuwamura M. Dietary iron overload enhances western diet induced hepatic inflammation and alters lipid metabolism in rats sharing similarity with human DIOS. Sci Rep. (2022) 12(1):21414. 10.1038/s41598-022-25838-3 36496443 PMC9741655

[59] Vinchi F Porto G Simmelbauer A Altamura S Passos ST Garbowski M Atherosclerosis is aggravated by iron overload and ameliorated by dietary and pharmacological iron restriction. Eur Heart J. (2020) 41(28):2681–95. 10.1093/eurheartj/ehz112 30903157

[60] Han M Guan L Ren Y Zhao Y Liu D Zhang D Dietary iron intake and risk of death due to cardiovascular diseases: a systematic review and dose-response meta-analysis of prospective cohort studies. Asia Pac J Clin Nutr. (2020) 29(2):309–21. 10.6133/apjcn.202007_29(2).0014 32674239

[61] Guo Z Ran Q 2nd Roberts LJ Zhou L Richardson A Sharan C Suppression of atherogenesis by overexpression of glutathione peroxidase-4 in apolipoprotein E-deficient mice. Free Radic Biol Med. (2008) 44(3):343–52. 10.1016/j.freeradbiomed.2007.09.009 18215741 PMC2245803

[62] Wu X Qin K Iroegbu CD Xiang K Peng J Guo J Genetic analysis of potential biomarkers and therapeutic targets in ferroptosis from coronary artery disease. J Cell Mol Med. (2022) 26(8):2177–90. 10.1111/jcmm.17239 35152560 PMC8995456

[63] He L Liu YY Wang K Li C Zhang W Li ZZ Tanshinone IIA protects human coronary artery endothelial cells from ferroptosis by activating the Nrf2 pathway. Biochem Biophys Res Commun. (2021) 575:1–7. 10.1016/j.bbrc.2021.08.067 34454174

[64] Yang K Song H Yin D. PDSS2 inhibits the ferroptosis of vascular endothelial cells in atherosclerosis by activating Nrf2. J Cardiovasc Pharmacol. (2021) 77(6):767–76. 10.1097/fjc.0000000000001030 33929387 PMC8274586

[65] Seferović PM Polovina M Bauersachs J Arad M Ben Gal T Lund LH Heart failure in cardiomyopathies: a position paper from the heart failure association of the European society of cardiology. Eur J Heart Fail. (2019) 21(5):553–76. 10.1002/ejhf.1461 30989768

[66] Li D Pi W Sun Z Liu X Jiang J. Ferroptosis and its role in cardiomyopathy. Biomed Pharmacother. (2022) 153:113279. 10.1016/j.biopha.2022.113279 35738177

[67] Wang Y Yan S Liu X Deng F Wang P Yang L PRMT4 promotes ferroptosis to aggravate doxorubicin-induced cardiomyopathy via inhibition of the Nrf2/GPX4 pathway. Cell Death Differ. (2022) 29(10):1982–95. 10.1038/s41418-022-00990-5 35383293 PMC9525272

[68] Ichikawa Y Ghanefar M Bayeva M Wu R Khechaduri A Naga Prasad SV Cardiotoxicity of doxorubicin is mediated through mitochondrial iron accumulation. J Clin Invest. (2014) 124(2):617–30. 10.1172/jci72931 24382354 PMC3904631

[69] Tadokoro T Ikeda M Ide T Deguchi H Ikeda S Okabe K Mitochondria-dependent ferroptosis plays a pivotal role in doxorubicin cardiotoxicity. JCI Insight. (2020) 5(9):e132747. 10.1172/jci.insight.132747 32376803 PMC7253028

[70] Kitakata H Endo J Matsushima H Yamamoto S Ikura H Hirai A MITOL/MARCH5 determines the susceptibility of cardiomyocytes to doxorubicin-induced ferroptosis by regulating GSH homeostasis. J Mol Cell Cardiol. (2021) 161:116–29. 10.1016/j.yjmcc.2021.08.006 34390730

[71] Meng L Lin H Huang X Weng J Peng F Wu S. METTL14 suppresses pyroptosis and diabetic cardiomyopathy by downregulating TINCR lncRNA. Cell Death Dis. (2022) 13(1):38. 10.1038/s41419-021-04484-z 35013106 PMC8748685

[72] Gong CW Yuan MM Qiu BQ Wang LJ Zou HX Hu T Identification and validation of ferroptosis-related biomarkers in septic cardiomyopathy via bioinformatics analysis. Front Genet. (2022) 13:827559. 10.3389/fgene.2022.827559 35495160 PMC9043284

[73] Kong C Ni X Wang Y Zhang A Zhang Y Lin F ICA69 aggravates ferroptosis causing septic cardiac dysfunction via STING trafficking. Cell Death Discov. (2022) 8(1):187. 10.1038/s41420-022-00957-y 35397620 PMC8994779

[74] Murtaza G Virk HUH Khalid M Lavie CJ Ventura H Mukherjee D Diabetic cardiomyopathy—a comprehensive updated review. Prog Cardiovasc Dis. (2019) 62(4):315–26. 10.1016/j.pcad.2019.03.003 30922976

[75] Zang H Wu W Qi L Tan W Nagarkatti P Nagarkatti M Autophagy inhibition enables Nrf2 to exaggerate the progression of diabetic cardiomyopathy in mice. Diabetes. (2020) 69(12):2720–34. 10.2337/db19-1176 32948607 PMC7679777

[76] Wang N Ma H Li J Meng C Zou J Wang H HSF1 functions as a key defender against palmitic acid-induced ferroptosis in cardiomyocytes. J Mol Cell Cardiol. (2021) 150:65–76. 10.1016/j.yjmcc.2020.10.010 33098823

[77] Zhang Z Tang J Song J Xie M Liu Y Dong Z Elabela alleviates ferroptosis, myocardial remodeling, fibrosis and heart dysfunction in hypertensive mice by modulating the IL-6/STAT3/GPX4 signaling. Free Radic Biol Med. (2022) 181:130–42. 10.1016/j.freeradbiomed.2022.01.020 35122997

[78] Fang X Cai Z Wang H Han D Cheng Q Zhang P Loss of cardiac ferritin H facilitates cardiomyopathy via Slc7a11-mediated ferroptosis. Circ Res. (2020) 127(4):486–501. 10.1161/circresaha.120.316509 32349646

[79] Zhang X Zheng C Gao Z Chen H Li K Wang L SLC7A11/xCT prevents cardiac hypertrophy by inhibiting ferroptosis. Cardiovasc Drugs Ther. (2022) 36(3):437–47. 10.1007/s10557-021-07220-z 34259984

[80] Menon AV Liu J Tsai HP Zeng L Yang S Asnani A Excess heme upregulates heme oxygenase 1 and promotes cardiac ferroptosis in mice with sickle cell disease. Blood. (2022) 139(6):936–41. 10.1182/blood.2020008455 34388243 PMC8832481

[81] Rose RA Sellan M Simpson JA Izaddoustdar F Cifelli C Panama BK Iron overload decreases CaV1.3-dependent L-type Ca2+ currents leading to bradycardia, altered electrical conduction, and atrial fibrillation. Circ Arrhythm Electrophysiol. (2011) 4(5):733–42. 10.1161/circep.110.960401 21747058 PMC3401539

[82] Sripetchwandee J KenKnight SB Sanit J Chattipakorn S Chattipakorn N. Blockade of mitochondrial calcium uniporter prevents cardiac mitochondrial dysfunction caused by iron overload. Acta Physiol (Oxf). (2014) 210(2):330–41. 10.1111/apha.12162 24034353

[83] Dai C Kong B Qin T Xiao Z Fang J Gong Y Inhibition of ferroptosis reduces susceptibility to frequent excessive alcohol consumption-induced atrial fibrillation. Toxicology. (2022) 465:153055. 10.1016/j.tox.2021.153055 34864093

[84] Li N Wang W Zhou H Wu Q Duan M Liu C Ferritinophagy-mediated ferroptosis is involved in sepsis-induced cardiac injury. Free Radic Biol Med. (2020) 160:303–18. 10.1016/j.freeradbiomed.2020.08.009 32846217

[85] Fang J Kong B Shuai W Xiao Z Dai C Qin T Ferroportin-mediated ferroptosis involved in new-onset atrial fibrillation with LPS-induced endotoxemia. Eur J Pharmacol. (2021) 913:174622. 10.1016/j.ejphar.2021.174622 34748769

[86] Zou HX Qiu BQ Lai SQ Zhou XL Gong CW Wang LJ Iron metabolism and idiopathic pulmonary arterial hypertension: new insights from bioinformatic analysis. Biomed Res Int. (2021) 2021:5669412. 10.1155/2021/5669412 34722766 PMC8556088

[87] Cotroneo E Ashek A Wang L Wharton J Dubois O Bozorgi S Iron homeostasis and pulmonary hypertension: iron deficiency leads to pulmonary vascular remodeling in the rat. Circ Res. (2015) 116(10):1680–90. 10.1161/circresaha.116.305265 25767292

[88] Hu P Xu Y Jiang Y Huang J Liu Y Wang D The mechanism of the imbalance between proliferation and ferroptosis in pulmonary artery smooth muscle cells based on the activation of SLC7A11. Eur J Pharmacol. (2022) 928:175093. 10.1016/j.ejphar.2022.175093 35700835

[89] Kung YA Chiang HJ Li ML Gong YN Chiu HP Hung CT Acyl-coenzyme a synthetase long-chain family member 4 is involved in viral replication organelle formation and facilitates virus replication via ferroptosis. mBio. (2022) 13(1):e0271721. 10.1128/mbio.02717-21 35038927 PMC8764547

[90] Zhang H Zhabyeyev P Wang S Oudit GY. Role of iron metabolism in heart failure: from iron deficiency to iron overload. Biochim Biophys Acta Mol Basis Dis. (2019) 1865(7):1925–37. 10.1016/j.bbadis.2018.08.030 31109456

[91] Chen X Xu S Zhao C Liu B. Role of TLR4/NADPH oxidase 4 pathway in promoting cell death through autophagy and ferroptosis during heart failure. Biochem Biophys Res Commun. (2019) 516(1):37–43. 10.1016/j.bbrc.2019.06.015 31196626

[92] Li W Feng G Gauthier JM Lokshina I Higashikubo R Evans S Ferroptotic cell death and TLR4/Trif signaling initiate neutrophil recruitment after heart transplantation. J Clin Invest. (2019) 129(6):2293–304. 10.1172/jci126428 30830879 PMC6546457

[93] Chakraborty A Li Y Zhang C Li Y LeMaire SA Shen YH. Programmed cell death in aortic aneurysm and dissection: a potential therapeutic target. J Mol Cell Cardiol. (2022) 163:67–80. 10.1016/j.yjmcc.2021.09.010 34597613 PMC8816882

[94] Ren J Lv Y Wu L Chen S Lei C Yang D Key ferroptosis-related genes in abdominal aortic aneurysm formation and rupture as determined by combining bioinformatics techniques. Front Cardiovasc Med. (2022) 9:875434. 10.3389/fcvm.2022.875434 36017103 PMC9395677

[95] Zhuang J Zhu H Cheng Z Hu X Yu X Li J PCSK9, a novel immune and ferroptosis related gene in abdominal aortic aneurysm neck. Sci Rep. (2023) 13(1):6054. 10.1038/s41598-023-33287-9 37055467 PMC10102181

[96] Li W Li W Leng Y Xiong Y Xia Z. Ferroptosis is involved in diabetes myocardial ischemia/reperfusion injury through endoplasmic reticulum stress. DNA Cell Biol. (2020) 39(2):210–25. 10.1089/dna.2019.5097 31809190

[97] Ning D Yang X Wang T Jiang Q Yu J Wang D. Atorvastatin treatment ameliorates cardiac function and remodeling induced by isoproterenol attack through mitigation of ferroptosis. Biochem Biophys Res Commun. (2021) 574:39–47. 10.1016/j.bbrc.2021.08.017 34438345

[98] Ma S He LL Zhang GR Zuo QJ Wang ZL Zhai JL Canagliflozin mitigates ferroptosis and ameliorates heart failure in rats with preserved ejection fraction. Naunyn Schmiedebergs Arch Pharmacol. (2022) 395(8):945–62. 10.1007/s00210-022-02243-1 35476142 PMC9276585

[99] Wang Z Yao M Jiang L Wang L Yang Y Wang Q Dexmedetomidine attenuates myocardial ischemia/reperfusion-induced ferroptosis via AMPK/GSK-3β/Nrf2 axis. Biomed Pharmacother. (2022) 154:113572. 10.1016/j.biopha.2022.113572 35988428

[100] Shan X Lv ZY Yin MJ Chen J Wang J Wu QN. The protective effect of cyanidin-3-glucoside on myocardial ischemia-reperfusion injury through ferroptosis. Oxid Med Cell Longev. (2021) 2021:8880141. 10.1155/2021/8880141 33628391 PMC7884153

[101] Sun X Sun P Zhen D Xu X Yang L Fu D Melatonin alleviates doxorubicin-induced mitochondrial oxidative damage and ferroptosis in cardiomyocytes by regulating yap expression. Toxicol Appl Pharmacol. (2022) 437:115902. 10.1016/j.taap.2022.115902 35093381

[102] Li T Tan Y Ouyang S He J Liu L. Resveratrol protects against myocardial ischemia-reperfusion injury via attenuating ferroptosis. Gene. (2022) 808:145968. 10.1016/j.gene.2021.145968 34530090

[103] Tang LJ Luo XJ Tu H Chen H Xiong XM Li NS Ferroptosis occurs in phase of reperfusion but not ischemia in rat heart following ischemia or ischemia/reperfusion. Naunyn Schmiedebergs Arch Pharmacol. (2021) 394(2):401–10. 10.1007/s00210-020-01932-z 32621060

[104] Lu H Xiao H Dai M Xue Y Zhao R. Britanin relieves ferroptosis-mediated myocardial ischaemia/reperfusion damage by upregulating GPX4 through activation of AMPK/GSK3β/Nrf2 signalling. Pharm Biol. (2022) 60(1):38–45. 10.1080/13880209.2021.2007269 34860639 PMC8648013

[105] Chen Y Yi X Huo B He Y Guo X Zhang Z BRD4770 functions as a novel ferroptosis inhibitor to protect against aortic dissection. Pharmacol Res. (2022) 177:106122. 10.1016/j.phrs.2022.106122 35149187

[106] Shen Y Shen X Wang S Zhang Y Wang Y Ding Y Protective effects of salvianolic acid B on rat ferroptosis in myocardial infarction through upregulating the Nrf2 signaling pathway. Int Immunopharmacol. (2022) 112:109257. 10.1016/j.intimp.2022.109257 36174419

[107] Chen L Liu Y Wang Z Zhang L Xu Y Li Y Mesenchymal stem cell-derived extracellular vesicles protect against abdominal aortic aneurysm formation by inhibiting net-induced ferroptosis. Exp Mol Med. (2023) 55(5):939–51. 10.1038/s12276-023-00986-2 37121969 PMC10238484

[108] Olivas-Aguirre FJ Rodrigo-García J Martínez-Ruiz ND Cárdenas-Robles AI Mendoza-Díaz SO Álvarez-Parrilla E Cyanidin-3-O-glucoside: physical-chemistry, foodomics and health effects. Molecules. (2016) 21(9):1264. 10.3390/molecules21091264 27657039 PMC6273591

[109] Kim SG Lee E Park NY Park HH Jeong KT Kim KJ Britanin attenuates ovalbumin-induced airway inflammation in a murine asthma model. Arch Pharmacal Res. (2016) 39(7):1006–12. 10.1007/s12272-016-0783-z 27342608

[110] Guo Z Lin J Sun K Guo J Yao X Wang G Deferoxamine alleviates osteoarthritis by inhibiting chondrocyte ferroptosis and activating the Nrf2 pathway. Front Pharmacol. (2022) 13:791376. 10.3389/fphar.2022.791376 35359876 PMC8964096

[111] Luo LF Guan P Qin LY Wang JX Wang N Ji ES. Astragaloside IV inhibits adriamycin-induced cardiac ferroptosis by enhancing Nrf2 signaling. Mol Cell Biochem. (2021) 476(7):2603–11. 10.1007/s11010-021-04112-6 33656642

[112] Li D Liu X Pi W Zhang Y Yu L Xu C Fisetin attenuates doxorubicin-induced cardiomyopathy in vivo and in vitro by inhibiting ferroptosis through SIRT1/Nrf2 signaling pathway activation. Front Pharmacol. (2021) 12:808480. 10.3389/fphar.2021.808480 35273493 PMC8902236

[113] Tadokoro T Ikeda M Abe K Ide T Miyamoto HD Furusawa S Ethoxyquin is a competent radical-trapping antioxidant for preventing ferroptosis in doxorubicin cardiotoxicity. J Cardiovasc Pharmacol. (2022) 80(5):690–9. 10.1097/fjc.0000000000001328 35881422

[114] Chen H Zhu J Le Y Pan J Liu Y Liu Z Salidroside inhibits doxorubicin-induced cardiomyopathy by modulating a ferroptosis-dependent pathway. Phytomedicine. (2022) 99:153964. 10.1016/j.phymed.2022.153964 35180677

[115] Chen Y Li S Yin M Li Y Chen C Zhang J Isorhapontigenin attenuates cardiac microvascular injury in diabetes via the inhibition of mitochondria-associated ferroptosis through PRDX2-MFN2-ACSL4 pathways. Diabetes. (2023) 72(3):389–404. 10.2337/db22-0553 36367849

[116] Naujokat C McKee DL. The “Big Five” phytochemicals targeting cancer stem cells: curcumin, EGCG, sulforaphane, resveratrol and genistein. Curr Med Chem. (2021) 28(22):4321–42. 10.2174/0929867327666200228110738 32107991

[117] Wang X Chen X Zhou W Men H Bao T Sun Y Ferroptosis is essential for diabetic cardiomyopathy and is prevented by sulforaphane via AMPK/NRF2 pathways. Acta Pharm Sin B. (2022) 12(2):708–22. 10.1016/j.apsb.2021.10.005 35256941 PMC8897044

[118] Wei Z Shaohuan Q Pinfang K Chao S. Curcumin attenuates ferroptosis-induced myocardial injury in diabetic cardiomyopathy through the Nrf2 pathway. Cardiovasc Ther. (2022) 2022:3159717. 10.1155/2022/3159717 35909950 PMC9307414

[119] Peng H Zhang J Zhang Z Turdi S Han X Liu Q Cardiac-specific overexpression of catalase attenuates lipopolysaccharide-induced cardiac anomalies through reconciliation of autophagy and ferroptosis. Life Sci. (2023) 328:121821. 10.1016/j.lfs.2023.121821 37257582

[120] Shen Y Wang X Shen X Wang Y Wang S Zhang Y Geniposide possesses the protective effect on myocardial injury by inhibiting oxidative stress and ferroptosis via activation of the Grsf1/GPx4 axis. Front Pharmacol. (2022) 13:879870. 10.3389/fphar.2022.879870 35600863 PMC9117627

[121] Packer M. Potential interactions when prescribing SGLT2 inhibitors and intravenous iron in combination in heart failure. JACC Heart Fail. (2023) 11(1):106–14. 10.1016/j.jchf.2022.10.004 36396554

